# Neuro‐XR Primer: Blending Neuroscience and Extended Reality to Facilitate Cognitive Investigation

**DOI:** 10.1111/ejn.70733

**Published:** 2026-10-28

**Authors:** Clíona L. Murray, Nicholas A. Alexander, Paola Pinti, Samantha E. A. Gregory, Ulrike Senftleben

**Affiliations:** ^1^ Department of Psychiatry Yale University New Haven Connecticut USA; ^2^ Functional Imaging Laboratory (FIL), Department of Imaging Neuroscience, UCL Queen Square Institute of Neurology University College London London UK; ^3^ Department of Psychological Sciences Birkbeck, University of London London UK; ^4^ University of Salford Salford UK; ^5^ TUD Dresden University of Technology Dresden Germany

**Keywords:** extended reality, mobile neuroimaging, neuroimaging, Neuro‐XR, virtual environments

## Abstract

Neuro‐XR refers to the amalgamation of neuroimaging and extended reality (XR) including augmented, mixed and virtual reality. Both sets of technology have developed rapidly and in isolation, and here, we show that they have great potential when combined. Mobile neuroimaging technology is becoming increasingly available, offering researchers greater flexibility. Similarly, XR devices are now affordable and lightweight and can be coupled with open‐source software for developing experiments. We are now at a time where neuroimaging and XR are paired to investigate new cognitive questions but also to replicate seminal research. Neuro‐XR provides cognitive neuroscience and XR‐based applications such as medicine, quantitative understanding (through behaviour and neural data) and analysis of real‐time interactions. In this primer, we introduce the term ‘Neuro‐XR’ and define the scope of the technique through a concept‐driven synthesis based on the literature and driven by the authors' expertise. More generally, we discuss the current research in the field and explore some potential future applications. Finally, we provide suggestions and guidance for practising Neuro‐XR based on our combined experience across these modalities.

## Graphical abstract

We present a comprehensive introduction and overview of the novel combination of neuroimaging and extended reality (XR), as Neuro‐XR. Three main combinations are explored throughout the manuscript: EEG‐XR, fNIRS‐XR and OP‐MEG‐XR. We provide specific guidance for developing Neuro‐XR experiments and broader considerations for the field.

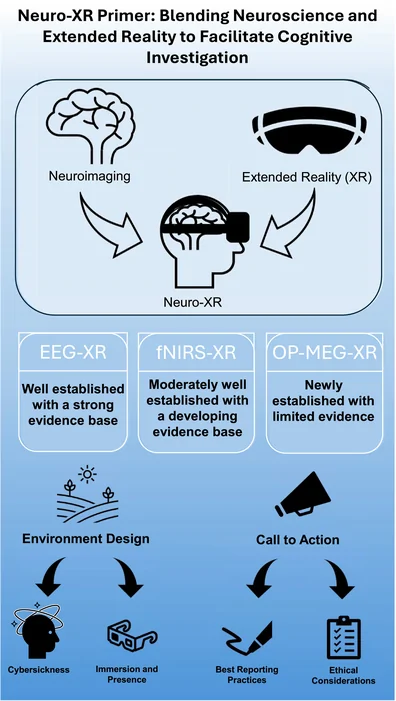

**Abbreviations**
2D: two dimensional
3D: three dimensional
AR: augmented reality
BOLD: blood oxygen level dependent response
CAVE: Cave Automatic Virtual Environment
EEG: electroencephalography
EEG‐XR: electroencephalography‐extended reality
ERP: event‐related potential
fMRI: functional magnetic resonance imaging
fNIRS: functional near infrared spectroscopy
fNIRS‐XR: functional near infrared spectroscopy‐extended reality
FOV: field of view
HMD: head‐mounted display
MEG: magnetoencephalography
MR: mixed reality
MSR: magnetically shielded‐room
Neuro‐XR: neuroimaging‐extended reality
OP‐MEG: optically pumped magnetoencephalography
OP‐MEG‐XR: optically pumped magnetoencephalography‐extended reality
PPIE: patient and public involvement and engagement
SPA‐fNIRS: systemic physiology augmented–functional near infrared spectroscopy
VH: virtual human
VR: virtual reality
VR‐HMD: virtual reality–head‐mounted display
XR: extended reality

## 1 Introduction

One of the main challenges in cognitive neuroscience is how to study brain and behaviour under naturalistic conditions. Typically, cognitive neuroscience research is conducted using lab‐based experiments where participants have to respond to simple, low‐dimensional stimuli. Often, participants' mobility is limited (e.g., staying still in a supine position in magnetic resonance imaging research). Such lab‐based studies have the great advantage of maximising experimental control and data quality, yielding high internal validity. However, this creates two major issues. First, lab‐based studies have limited assumed ecological validity. This means we cannot be sure if the same behaviour and brain processes observed in the lab also occur under realistic circumstances. Second, there are phenomena that can only be studied using complex, naturalistic scenarios (e.g., autobiographical memory; Maguire 2022). These research questions are ignored in typical lab‐based cognitive neuroscience studies and hence remain unanswered.

Therefore, it is crucial to find a new approach for naturalistic, complex cognitive neuroscience studies. Due to recent technological advancements, neuroimaging has become increasingly mobile, allowing recording of brain activity while participants are moving. At the same time, it has become possible to present virtual stimuli that are complex and natural in appearance using extended reality (XR) technology. Combining neuroimaging methods with XR (an approach we call Neuro‐XR) allows researchers to record brain activity in complex, naturalistic scenarios that still offer experimental control. Here, we present evidence that Neuro‐XR is a promising tool for the future of cognitive neuroscience.

XR is an umbrella term that refers to a range of technologies that display 2D and 3D computer‐generated stimuli to the user on a spectrum of virtual and real environments (Rauschnabel et al. 2022; Box 1). This spectrum encapsulates environments ranging from real‐life, to technologies like augmented reality (AR) and virtual reality (VR). Although a strict definition has not been widely adopted within the field, in this paper, we take an applied, contemporary definition of VR as an experimental scene that has been built using 3D software (e.g., unity or unreal engine; see Table S1) and displayed using the current immersive hardware that are available such as VR headsets and CAVEs (Cave Automatic Virtual Environment).

The potential of XR can be seen across cognitive psychology and cognitive neuroscience. For example, XR has opened up new ways to study effects such as the material‐weight illusion (Naylor et al. 2022), the size‐weight illusion (Buckingham 2019; Rohrbach et al. 2021) and motor cognition during complex movement, facilitated by the motion‐tracking capabilities of XR (for a review, see Buckingham 2021). Further, XR makes it possible to test interactions with human‐like virtual agents, thus maintaining experimental control while presenting socially relevant stimuli (Pan and Hamilton 2018). XR has also been used to study real world cognition using safe, affordable simulations of real‐life settings (e.g., driving simulations; Bennett et al. 2016; Ihemedu‐Steinke et al. 2015, 2017; Senftleben and Kessler 2024; Taheri et al. 2017).

Crucially, the early combination of neuroimaging and XR has been pivotal in both academic and clinical settings. Neuro‐XR is versatile, relatively inexpensive and mobile. The dominant, most prevalent mobile neuroimaging techniques available are electroencephalography (EEG), functional near infrared spectroscopy (fNIRS) and optically pumped magnetometer (OPM)–based magnetoencephalography (OP‐MEG; see Table 1 for overview). Currently, EEG and fNIRS are relatively affordable (~£/$10,000–60,000; this may vary based on configuration, region and time) and pair well with XR, whereas OPMs are comparatively early in their development for regular use as a cognitive neuroscience technique. Fortunately, due to the use in gaming, XR hardware has become affordable and commercially available, whereas powerful software for the creation of XR environments is freely available and well supported.

**TABLE 1:** An adaption of tab. 1 in Richer et al. (2024), displaying an overview of each of the Neuro‐XR combinations in relation to the technical considerations for research.

Neuro‐XR tool	What it measures	Temporal resolution	Spatial resolution	Tolerance to movement	Compatibility with XR	Main sources of artefacts	How established is the technique	Types of questions that are best suited
EEG‐XR	Electrical potentials	Millisecond range	Centimetre range, improved with MRI coregistration	Movement causes significant noise in signal	Virtual reality, augmented reality and mixed reality	Muscle movement, eye blinks, cable sway, facial movements (jaw/chewing)	Very well established with a strong evidence base	‘When?’ questions
fNIRS‐XR	Relative changes in blood oxygenation and haemodynamics	Second range	Centimetre range, restricted to areas near the scalp	It is relatively robust to head movements and walking	Virtual reality, augmented reality and mixed reality	Systemic changes, Optodes decoupling due to movement	Moderately well established with a developing evidence base	‘Where?’ (limited to cortical areas)
OP‐MEG‐XR	Magnetic fields produced by neuronal currents	Millisecond range	Millimetre range	It is robust to head movements. Walking is limited to the magnetically shielded room	All methods require significant modification to the XR headsets to be suitable for the magnetically shielded room.	Sensor motion, external metallic objects, external system interference	Newly established with limited evidence	‘When?’ and ‘where?’

In this paper, we present best practices of Neuro‐XR focusing on EEG, fNIRS and OP‐MEG. We take a narrative, concept‐driven synthesis approach based on the authors' expertise and current literature, intended to guide researchers in evaluating when and how to adopt Neuro‐XR techniques. We will provide suggestions on ways to use Neuro‐XR to its full capacity while maintaining ecological validity and experimental control. We will also discuss potential pitfalls and highlight scenarios where Neuro‐XR may not be the best investigative choice. By doing this, we will enable researchers to consider factors related to their research question, their experimental design and subsequent adoption (or not) of the Neuro‐XR technique method.

## 2 Neuro‐XR Approaches

First, we will briefly outline key research design principles applicable across Neuro‐XR techniques, and then, we will detail each Neuro‐XR approach, outlining specific challenges. This will include an explanation of structured and unstructured designs and their implications for Neuro‐XR research as well as the importance of timing precision for designing Neuro‐XR studies.

### 2.1 Research Design Considerations

Structured designs are often experimental in nature and aim to capture specific behaviours or processes of interest in a systematic, controlled manner. This is typically achieved using multiple trials in which stimuli are presented, and responses are required at specific times. Trials of different conditions may either be presented randomly or in a blocked manner. Data from structured designs can be analysed using conventional analysis approaches, which are often time‐locked in nature (e.g., Luck et al. 2000). This is possible due to the repetitive controlled trial structure that is typically used. Structured designs present the gold standard for experimental research, often to obtain high statistical power, but this rigid structure can limit the research questions that can be answered. For example, complex, naturalistic behaviours might be difficult to capture using such highly controlled designs.

An alternative research approach is unstructured designs. These are often observational in nature. Instead of having a trial structure, they rely on a continuous (single‐trial) experience where behaviours or processes of interest may arise at different times (e.g., a participant freely exploring a city). This allows for more naturalistic behaviours and more complex research questions to be asked. Consequently, researchers have little or no control over when or how often a certain behaviour occurs, which might limit statistical power due to signal‐to‐noise ratio. Due to the uncertainty of events, unstructured designs typically require more complex data analysis approaches (e.g., Crosse et al. 2016; Maselli et al. 2023; O'Neill et al. 2025; Schneider et al. 2023).

Both structured and unstructured studies often rely on timing accuracy (e.g., stimulus presentation, recording of movements, recording of behavioural and neuroimaging data). This requires event triggers that are sent from the XR system to the neuroimaging system. In a structured study design, these triggers would be informative of specific events during a trial (e.g., trial onset and stimulus presentation), as well as a participant's response to these events. In an unstructured study design, triggers could be programmed to occur when participants interact with certain objects or when they pass certain markers in an environment, as well as being linked to participant response.

However, XR system capabilities pose potential challenges to the temporal precision of these event triggers. These include refresh rates, sampling rates and latencies caused by software and hardware. Refresh rates are fixed in XR headsets (typically 60–120 frames per second), which might limit stimulus presentation (especially in studies relying on fast‐changing stimuli). The frame rate might also drop if performance demands are too high, leading to reprojected or dropped frames (Wiesing et al. 2020; also see Building XR environments). Sampling rates can also affect timing precision. For example, some XR controllers have fixed sampling rates well below the typical sampling rates in keyboard‐based computer experiments, which limits the precision of response measurements (Wiesing et al. 2020). In addition, XR hardware and software can contain latencies that researchers need to be aware of. First, rendering pipelines implemented in typical XR software may induce latencies that can affect stimulus and response timing (Wiesing et al. 2020). Second, XR devices must consider motion‐to‐photon latency, which is the lag between a participant's movement and the corresponding change in the display. Motion‐to‐photon latency ranges from 21 to 42 ms and is particularly problematic for sudden, large movements (Warburton et al. 2023). In face of these issues, it is important to evaluate the temporal precision of Neuro‐XR setups. This can be done by measuring different types of latencies (Stauffert et al. 2020; Warburton et al. 2023; Wiesing et al. 2020). Furthermore, using software solutions such as the Unity Experimental Framework (Brookes et al. 2020) can help mitigate some issues with latency.

### 2.2 EEG‐XR

Electroencephalography (EEG) measures electrical activity generated by the brain, allowing researchers to make inferences about how the brain responds to specific stimuli. EEG has high temporal resolution in the millisecond range, enabling us to study the timing of cognitive processes in the brain. However, spatial resolution is poor; thus, it is more difficult to link observed activity to specific brain regions which support distinct neural processes. Broadly, EEG‐XR researchers approach data collection and analyses from two scenarios: either traditional, contrived experimental designs; or continuous, observational tasks. Traditional experimental designs are often analysed through methods that are complimentary of time‐locked designs, such as event related potentials (ERPs) (see Sur and Sinha 2009), or event‐related synchronisation and desynchronisation of rhythmic activity (see Herrmann et al. 2016; Pfurtscheller and Lopes da Silva 1999). Both ERPs and rhythmic activity reflect synchronised activity of aligned neuronal populations in response to neural events, with modulation of these signals being thought to reflect different psychological states (see Rangaswamy and Porjesz 2008). Alternatively, observational tasks could be analysed using techniques based on ongoing rhythmic activity such as connectivity analysis (Zhang et al. 2023).

Compared to other neuroimaging methods such as functional magnetic resonance imaging (fMRI), EEG is a highly cost‐effective solution for measuring neural effects of stimuli. Although EEG systems benefit from the use of a Faraday cage to shield the system from electrical noise, contemporary EEG systems make use of shielded electrodes and/or cables, increasing their versatility to high noise environments. Importantly for EEG‐XR, wireless, battery‐powered amplifiers are available, offering flexible usage such as allowing participants to explore XR environments freely. Hence, EEG pairs well with immersive XR in terms of head‐mounted displays (HMD), as well as setups like the CAVEs and screen‐based XR, allowing for greater realism.

Because of these advancements, EEG and XR can be readily combined without any specific development required, which has resulted in an abundance of EEG‐XR research. This varies across domains, with research looking at emotional cognitive factors (el Basbasse et al. 2023), social factors (Gregory et al. 2021; Kelly et al. 2020), factors involved in driving (Senftleben and Kessler 2024; Huizeling et al. 2020) and decision making (Alexander et al. 2024). When investigating phenomena using EEG‐XR, it is important to consider the potential issues arising from this pairing. These include timing precision, the interfering effects of the XR equipment on signal quality and the introduction of movement‐based artefacts.

The first issue to consider is timing precision. Although investigations using oscillatory activity are more forgiving of small inconsistencies in timing (~10 ms), ERP studies rely on precisely timed stimulus presentation due to phase‐locking. Any nonphase‐locked EEG activity is averaged out. Therefore, precise timing and alignment to EEG is extremely important. Any variable latencies in stimulus presentation or response measurement (as discussed above in Research Design Considerations) pose a challenge for ERP analyses. Hence, it is important to consider whether the XR system used supports the temporal precision required by the research question. If not, the advantages of high temporal precision in EEG are lost. Solutions may involve an adaptation to the XR technology or a different method.

The next issue to consider is interference from the XR equipment on the EEG signal. A number of researchers have assessed the impact of XR headsets on signal quality in EEG (e.g., Cattan et al. 2018; Hertweck et al. 2019; Tauscher et al. 2019; Weber et al. 2021). These studies show that although the headsets produce artefacts, these are typically limited to line noise and the display's refresh rate. This is a result of close proximate electromagnetic interference that is picked up by the electrode leads and should have little impact on ERP or measurements of band‐limited power in lower frequencies. However, those interested in higher frequency activity such as gamma band may face difficulties. Additionally, extra consideration should be given to the straps of the headset. HMDs have straps to hold the system on the participants head, which cover electrode sites (see Figure 1). This can affect signal quality in these regions, for example, by causing movement of the cap in these locations when straps are tightened or when caps are put on or removed during breaks. However, some researchers have adapted headsets by changing the straps (el Basbasse et al. 2023) and removing earphones (Alexander et al. 2024) to avoid this issue.

**FIGURE 1:**
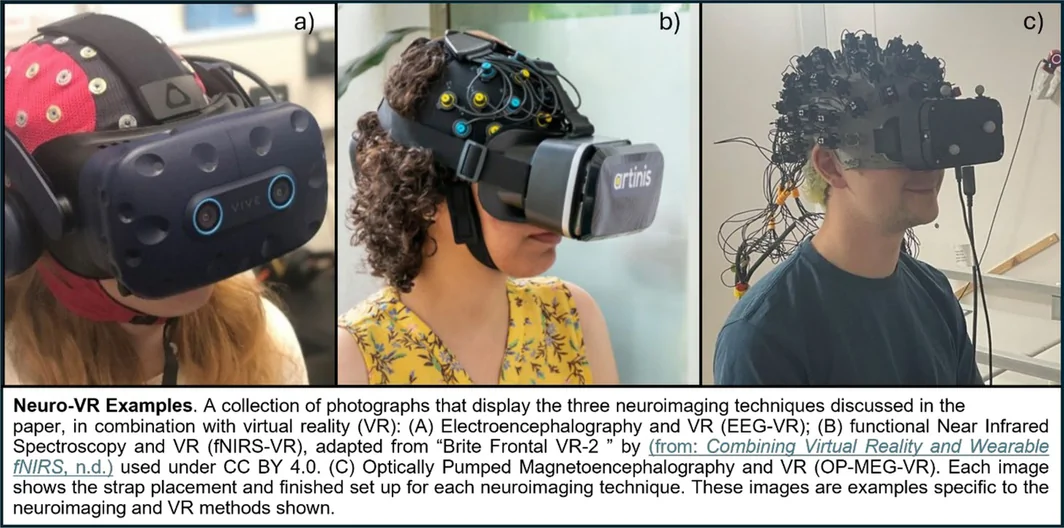
Neuro‐VR examples. A collection of photographs that display the three neuroimaging techniques discussed in the paper, in combination with virtual reality (VR): (A) electroencephalography and VR (EEG‐VR); (B) functional Near Infrared Spectroscopy and VR (fNIRS‐VR), adapted from ‘Brite Frontal VR‐2’ (from *Combining Virtual Reality and Wearable fNIRS*, n.d.) used under CC BY 4.0. (C) Optically pumped magnetoencephalography and VR (OP‐MEG‐VR). Each image shows the strap placement and finished set up for each neuroimaging technique. These images are examples specific to the neuroimaging and VR methods shown.

Often the purpose of using XR is to add realism to the research scenario, including allowing participants to explore the virtual environment both visually and physically. This brings us to our final issue of movement artefacts from the eyes and muscles. Eye movement artefacts are EEG potentials unrelated to neural events caused by interference from the contraction of the eye muscles (see Iwasaki et al. 2005; Plöchl et al. 2012). These artefacts are problematic as they are larger in amplitude than the neural signals of interest, and, critically, they are temporally linked. They could therefore obscure true signals of interest. Body movement also causes artefacts through muscle activity, as well as the rhythmic signals produced through activities such as walking (e.g., Gwin et al. 2011) that can also result in cable sway motion artefacts (Symeonidou et al. 2018). Current EEG preprocessing pipelines include processes for dealing with artefacts by either discarding noisy trials or cleaning them through statistical methods (e.g., independent component analysis; Klug and Gramann 2021). However, these preprocessing steps have been developed for and mainly used in highly controlled experiments designed to limit movement. Therefore, new methods are continuously being developed to deal with the large‐scale artefacts expected in XR. These require information around movement during experimental testing (i.e., head, body and eye movement) to ensure that solutions can be applied effectively (Gorjan et al. 2022). Hardware adaptions, such as dual‐sided EEG (Li and Huang 2025), have attempted to tackle the effects of movement on EEG signal and could inform improvements on data analysis. Modern XR tools often provide tracking of these aspects including eye tracking and full body motion capture, and these should be integrated, where possible, into preprocessing pipelines. However, any artefact correction method will risk signal loss, and this issue requires targeted development to tackle movement artefacts in EEG‐XR.

Therefore, when designing research using EEG with XR, it is important to consider your use case. Do you need highly precise timing, and to what degree is this feasible? Can your study design tolerate data loss due to movement artefacts? Is using a head‐mounted XR device going to add something beyond that which is possible using other XR devices, or even traditional screen‐based methods?

### 2.3 fNIRS‐XR

Functional near‐infrared spectroscopy (fNIRS) is an optical neuroimaging technology that measures the changes in blood oxygenation and haemodynamics in the brain. Similar to fMRI, fNIRS is based on neurovascular coupling. fNIRS estimates changes in concentration of oxygenated and deoxygenated haemoglobin by taking advantage of the different absorption properties of haemoglobin in the near infrared range (Scholkmann et al. 2014). This is achieved by placing optodes, which detect the light that is scattered back to the scalp by oxygenated and deoxygenated haemoglobin. Because blood oxygenation levels systematically vary with neuronal activity, this allows an indirect measure of neuronal processes (Villringer and Dirnagl 1995).

In cognitive neuroscience, fNIRS is typically used to measure localised changes in brain activity in reaction to specific stimuli or the functional connection across multiple brain regions. It can record activity from the cortex with a spatial resolution of 1.5–2 cm and a temporal resolution of 10 ms (Ferrari and Quaresima 2012; Pinti et al. 2020). Whereas the spatial resolution is inferior to fMRI and the brain coverage depends on the hardware and number of optodes available, fNIRS is relatively inexpensive and tolerant to body movements, portable (including wireless and increasingly lightweight options) and allows participants to move freely (Peng et al. 2024; Pinti et al. 2018; Scholkmann et al. 2014). Thus, fNIRS has potential for naturalistic experiments and would be expected to pair well with XR.

In comparison to EEG, fNIRS has a higher spatial resolution allowing researchers to localise brain activity with greater precision within the cortex. Unlike most EEG systems, fNIRS is also quick and easy to set up as it does not require gel. This allows greater accessibility for participant groups who would not always tolerate the arduous set up associated with EEG, for example, some clinical populations. Overall, fNIRS is robust to muscle and motion artefacts (however, see below for potential motion‐related issues) and is not affected by electromagnetic interference. This makes it well suited for use in situations that require a larger degree of body movements, for example, in immersive CAVEs with participants navigating freely in the virtual space (Bulgarelli et al. 2023; Pinti et al. 2024). For instance, fNIRS has been used successfully to record brain activity even when participants are engaged in motion‐intensive tasks such as cycling (Piper et al. 2014), table tennis (Balardin et al. 2017), basketball dribbling (Kanatschnig et al. 2021), playing an instrument (Balardin et al. 2017) or acting in a theatre performance (Greaves et al. 2022). This is an important advantage for studies that require motion in XR and one of the most promising avenues of combining fNIRS with XR.

fNIRS‐XR is still relatively rare and is still growing its community of practice. For instance, a recent review by Peng et al. (2024) highlighted that the combination of fNIRS with immersive VR is relatively new with only 37 papers published at the time of publication. Currently, fNIRS has been paired successfully with HMD‐based VR to investigate research areas such as social cognition (Kim et al. 2019), prospective memory (Dong et al. 2017, 2019) and the effect of stress and mental workload on task performance (Putze et al. 2019; Shi et al. 2020). Additionally, fNIRS has been combined with AR‐systems like the Hololens or Google Glass to investigate its feasibility in brain‐computer interaction (Benitez‐Andonegui et al. 2020; Hu et al. 2019; McKendrick et al. 2016). More recently, Bulgarelli et al. (2023) and Pinti et al. (2024) have showcased the use of mobile fNIRS in combination with an immersive VR CAVE to investigate social development in young children. Although these studies show that it is possible to use fNIRS in XR settings, they come with limitations such as low sample size as well as a lack of preregistration and replication. When compared to the more established techniques of EEG and fMRI, a consensus and standard practices are yet to be achieved (see Yücel et al. 2021 on how the community is targeting this issue). Further, relatively little research has been carried out on how the combination of fNIRS with various types of XR devices and settings affects signal quality (Peng et al. 2024). These issues highlight the importance of developing a community of practice to harness the full potential of fNIRS‐XR.

There are some drawbacks to fNIRS and its combination with XR that need to be considered. These relate to movement, hardware and task design. We detail these issues and potential solutions below.

fNIRS signals are impacted by physiological processes that occur during movement, such as those regulating vascular tone and blood flow, both in the extracerebral and cerebral compartments of the head (e.g., blood pressure, PaCO2, heart rate and respiration; Tachtsidis and Scholkmann 2016). Therefore, excessive movements can lead to false positives and/or false negatives when inferring brain activity (Pinti et al. 2018). Several approaches have been proposed to minimise this issue, such as matching the amount of movement across task conditions (Pinti et al. 2023) measuring physiological signals alongside fNIRS (i.e., systemic physiology augmented fNIRS [SPA‐fNIRS]; Scholkmann et al. 2022), which then can be regressed out. A common approach is to use short separation channels (i.e., channels created by placing a source and a detector very close to each other, < 1 cm), which are sensitive to extracerebral changes to remove superficial contamination from the long separation channels (Yücel et al. 2015). Recently, Pinti et al. (2024) have shown a successful implementation of short separation channels by superficial signal regression to improve the localisation of fNIRS signals recorded in freely moving preschoolers (Pinti et al. 2024).

As with EEG, when combining fNIRS with XR devices, issues can also arise due to the placement of HMD straps as well as interference from exterior light sources. First, the position of HMD straps limits fNIRS emitter‐detector layouts, especially when optodes are located over the frontopolar cortex (see Figure 1). This might require customisation of the HMD and the fNIRS to ensure that all brain regions of interest can be accessed (e.g., Kim et al. 2019; Seraglia et al. 2011) and may result in loss of coverage. HMDs could also affect the coupling of the optodes on the scalp, distorting their signal. Second, when infrared‐based devices such as motion tracking and eye‐tracking are used in an XR setup, the infrared light from the cameras may interfere with the detectors of the fNIRS devices. In CAVEs, the use of bright screens could saturate the fNIRS detectors (Peng et al. 2024). However, some fNIRS devices incorporate a reference detector that is used to correct for ambient light (Pinti et al. 2018), or the problem can be mitigated by protecting the sensors, for example, using aluminium foil (McKendrick et al. 2017).

When designing tasks for fNIRS‐XR, the lower temporal resolution and the temporal lag of the BOLD response measured by fNIRS need to be considered. Structured, blocked designs are recommended over event‐related designs, whereby several repetitions of the same stimulus condition are presented within one block and compared to a baseline or other condition blocks. This prevents overlap of responses to different task conditions, which occurs in event‐related designs where stimuli from different task conditions are presented interleaved within one block (for more discussion on this topic, see Petersen and Dubis 2012). In order to also make use of unstructured designs that more closely resemble real‐world demands, new analysis tools and improved sensitivity of fNIRS sensors are required (Pinti et al. 2018).

Therefore, as with EEG‐XR, when designing research using fNIRS XR, it is important to consider your use case. Is your question better suited to a block or event‐related design? Are you planning to use external equipment that may interfere with the device? Will the limitations of HMD straps interfere with your regions of interest? Have you considered the impact of movement in your task design?

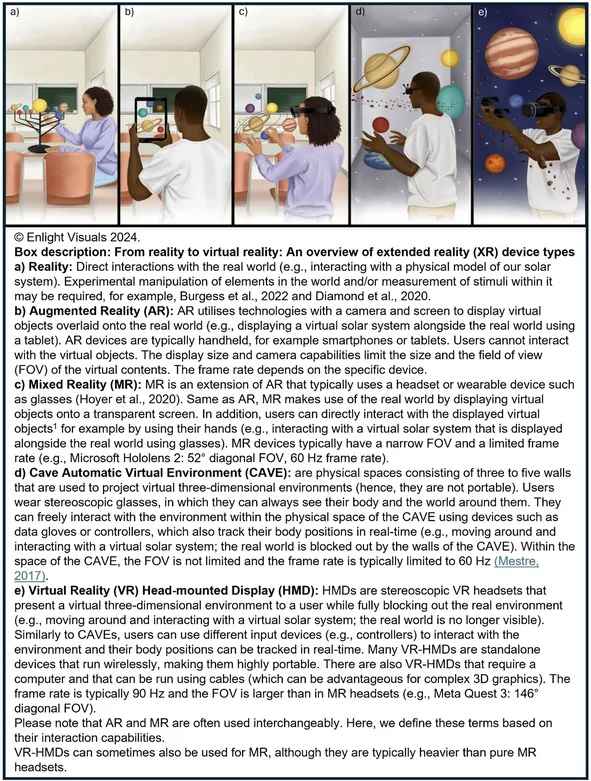

### 2.4 OP‐MEG‐XR

EEG and fNIRS, described above, represent a common trade‐off between spatial and temporal resolution in neuroimaging. Magnetoencephalography (MEG) is a noninvasive technique that offers both good spatial resolution at ~3–5 mm (Barratt et al. 2018) and sub‐millisecond temporal resolution (Baillet 2017). Most MEG systems use sensitive superconducting quantum interference devices (SQUIDs; Hämäläinen et al. 1993) to detect magnetic fields arising from the brain. However, SQUIDs require cryogenic cooling and sensors are held in a fixed array within an immobile helmet‐like device. Therefore, SQUID‐MEG is not portable and does not allow participants to move, making it suboptimal for XR research.

Development and miniaturisation of optically pumped magnetometer (OP‐MEG) sensors has enabled the production of wearable MEG systems (Boto et al. 2017). OPMs do not require cryogenic cooling (Brookes et al. 2022; Roberts et al. 2019), and individual sensors can be positioned in wearable arrays close to the scalp (Boto et al. 2019; Iivanainen et al. 2017), irrespective of participants' head shape or size. OP‐MEG has been demonstrated to have similar or improved signal‐to‐noise versus SQUID‐based MEG due to its increased proximity to the signal sources in the brain (Gaetz et al. 2026; Hill et al. 2020). Like SQUID‐MEG, OP‐MEG must be operated within a magnetically shielded room. In addition to the passive shielding provided by the room, active shielding may be used (Holmes et al. 2018; Mellor et al. 2023), and sensors may be operated in closed loop mode to support larger movements through the remnant field inside the room. Importantly, OP‐MEG has also been found to be more robust to muscle artefacts caused by movement in comparison to EEG (Boto et al. 2019). Altogether, this positions OP‐MEG as a promising neuroimaging method for movement‐based experimental paradigms, and beyond that, a method that has the potential to be combined with XR devices.

Though a promising combination with XR, OPMs are sensitive to magnetic fields produced by sources within the environment. Electrical equipment required to present stimuli, such as visual displays and speakers, is a significant source of electromagnetic interference. Therefore, commercially available XR devices, such as HMDs, are not an out‐of‐the‐box solution. Nevertheless, OP‐MEG has been combined with a modified commercial HMD (Roberts et al. 2019; see Figure 1) and typical neural signatures related to visual stimuli, specifically modulation in alpha amplitudes and the visual evoked field were detected. More recently, a bespoke, low‐metal HMD was combined with rigid, individually tailored helmets to support whole‐brain imaging (Zabbah et al. 2026). Despite these promising results, further demonstration of OP‐MEG‐XR is required and the authors discuss important limitations that need to be resolved.

Similar to early EEG models which required a Faraday cage, the use of OP‐MEG requires control of external magnetic fields (i.e., magnetic fields not caused by participants' brain activity), which can be achieved using coils within a magnetically shielded room (MSR). This places limitations on the size of the space in which participants can move. This also impacts the feasibility of alternatives to HMDs such as CAVEs. However, recent developments in active shielding support operation of OPMs without passive shielding (Bezsudnova et al. 2026). By removing the occluding walls, a CAVE system becomes viable. Further, these room‐free systems have been used with handheld electronic devices (Zheng et al. 2026), supporting the feasibility of HMDs and other XR devices. Despite these advances, substantial investment is required to successfully pair OP‐MEG and XR. This must cover the system itself, magnetic shielding, motion capture, custom electronics and task design, making this a challenge best tackled by an interdisciplinary team working in a specialised lab.

Theoretically, OP‐MEG is positioned as an optimal method for Neuro‐XR, but further development is required. Currently, OP‐MEG is not commonplace across neuroscience labs and institutions and has a high initial setup cost. This means that although the combination with XR is promising, it is not currently a practical alternative to EEG and fNIRS for most researchers. However, parallel developments in Neuro‐XR will progress uptake across all modalities by providing researchers with the tools to conduct experiments and collect data with XR methods.

### 2.5 Summary

EEG, fNIRS and OP‐MEGs have all been successfully paired with XR, with EEG‐XR currently representing the most mature and widely used approach. FNIRS‐XR is emerging as a promising but less standardised combination, offering greater tolerance to movement, whereas OP‐MEG‐XR remains in early development as its technical requirements are still being refined. Of the labs that are using Neuro‐XR, most published research has combined neuroimaging methods with VR HMDs (see Figure 1 for Neuro‐VR examples), reflecting the current accessibility and affordability of these systems. Importantly, XR headsets typically pair well with both fNIRS and EEG with the decisions around which to use mostly focused on the research question. However, it is important to consider how the headset straps of XR devices may impact the electrodes and optodes, as well as considerations around participant movement. Additionally, CAVE setups, and others using infrared light, for example, for motion tracking, may be better paired with EEG than with fNIRS due to potential interference.

## 3 Building XR Environments

Although it is important to consider the neuroimaging methods on offer and how they pair with XR, it is equally important to consider how to build a suitable virtual environment (see Vasser and Aru 2020). Depending on research question, researcher skillset, hardware and resources, there are many different software that can be used to build and host experiments. Free, commercially available game engines such as Unity (Unity3D 2019) and Unreal (Epic Games 2019) are commonly used, as well as software built for academic research such as Vizard (WorldViz 2008). Assets such as 3D objects including virtual humans and environments are available (some may require licences or payment) or they can be built from scratch using 3D modelling software for example, Blender (B. O. Community 2018). When considering what software to use it is important to consider the principles of open science (see Center for Open Science for more). It is important to use applications that allow other researchers to freely view or even clone the experiments designed in order to ensure research is as open as possible. A comprehensive list of resources on both hardware and software, and latest news on XR can be found in this GitHub. In Table S1, we have provided some key resources on environment design. Here we focus on the design choices that can be made in creating virtual environments for Neuro‐XR research. Three overarching elements should be considered: (i) immersion and presence, (ii) cybersickness and (iii) the role of realism and complexity. In this section, we will describe and detail the significance of each of these overarching design choices and provide insight on best practice for those who are new to Neuro‐XR.

### 3.1 Immersion and Presence

Immersion and presence are two terms that are frequently discussed when designing XR environments. Immersion refers to the quality of sensory information that an XR system is able to provide (e.g., graphics and haptics). Presence refers to the subjective engagement of an XR user (i.e., the feeling of ‘being there’). See Berkman and Akan (2019) for a comprehensive overview of these terms.

Immersion relies on the physical capabilities of the device to display the virtual environment. Therefore, it is important to know the performance capabilities (software and hardware) of your computer and XR device and use these to guide the design choices of your experiment. One aspect to consider is field of view (FOV): Clear or defined boundaries between the virtual and real world are less immersive than those that fill a user's FOV. For example, most modern VR headsets are built to maximise the users' FOV, whereas current MR headsets have a limited FOV in which virtual objects can be displayed (see Box 1). Similarly, software capabilities can limit the immersion; for example, complex virtual worlds can overwhelm computer and device capabilities leading to a lag in the environment. In conclusion, to maintain high immersion, we recommend that experiments are designed to be displayed as seamlessly as possible, with this being affected through simple design.

Presence is defined as the sense of being in an environment (Steuer 1992); in XR, we consider this as a subjective sense of ‘being there’ in the virtual world (Berkman and Akan 2019; Sanchez‐Vives and Slater 2005). This definition relies heavily on the participants' subjective experience of XR, which can be modulated by their knowledge and motivation (automatic and controlled mental processes, respectively; Gibson 2014; Steuer 1992) to believe the scenario. How immersive the environment is may facilitate an individual's sense of presence (Berkman and Akan 2019). Importantly, participants can feel a high sense of presence even if the environment is not realistic. Instead, presence can be strengthened by developing scenes that are engaging (Garrido et al. 2022) and artistically consistent and allow natural interaction (Lim and Ji 2023). Presence can therefore be affected by the nature of the interaction with the environment (Steuer 1992) such as immediate visual or haptic feedback, but a high degree of immersion does not automatically create presence.

In summary, there are many subjective (mental states) and objective (physical capabilities) parameters that can influence the user's experience within the virtual world.

### 3.2 Cybersickness

One unfortunate byproduct of XR design that can disrupt user experience is cybersickness. Also referred to as VR, motion or simulator sickness, symptoms include nausea, dizziness, cold sweats and eye strain directly caused by exposure to XR environments (LaViola 2000). According to the sensory conflict theory (Oman 1998), this occurs because of a sensory mismatch between what is perceived in the virtual world and what is perceived in the real world. Cybersickness is a significant issue for Neuro‐XR researchers, affecting behaviour and processes of interest, as well as causing high dropout rates (15%–20% reported; e.g., Reed et al. 2007; Saredakis et al. 2020). Cybersickness reduces presence and enjoyment of the virtual environment (Garrido et al. 2022). Therefore, it is important to consider the factors of XR environment design that can influence the levels of cybersickness in your study. These are (i) XR device type (e.g., HMD, glasses or handheld device), (ii) the content of the XR environment itself (realism and movement of stimuli), and (iii) the mode of navigation.

Cybersickness has been reported for almost all device types, from CAVEs (Kwok et al. 2018; Martirosov et al. 2022) and HMDs (Saredakis et al. 2020; Tian et al. 2022) to AR and MR‐devices (Hughes et al. 2020; Vovk et al. 2018; Zhang et al. 2024). However, cybersickness severity is typically larger in HMDs and CAVEs (Vovk et al. 2018). This is because these setups capture the user's complete FOV (see Box 1), which maximises the sensory mismatch between the virtual and real world, although there is inconsistent evidence if cybersickness symptoms are worse in HMDs or in CAVEs (Kwok et al. 2018; Martirosov et al. 2022). An important aspect to consider is that CAVE glasses allow users to see their own body, whereas HMDs do not. This might reduce cybersickness given indirect evidence from the use of HMDs: Using an avatar to represent one's own body has been found to reduce cybersickness (Makani et al. 2024). Further, cybersickness symptoms in XR can be decreased by limiting the amount of information presented. For HMDs, this can be achieved by reducing the virtual FOV or employing blurring in peripheral zones, mimicking how the human eye naturally views the world (Tian et al. 2022). In comparison, current AR and MR devices have a reduced FOV (see Box 1) and are therefore less likely to cause cybersickness (e.g., Hughes et al. 2020; Vovk et al. 2018), but note that this might also reduce immersion and presence as explained above.

Cybersickness is more likely to be an issue if the virtual scene contains movement. This can either be because individual stimuli in the environment are moving (local movement) or because the environment is moving (global movement). For example, when investigating driving, a driving simulation could involve local movement external to the vehicle such as other cars, the sky and pedestrians, as well as global movement due to the car moving through the environment. In these scenarios, the participant could be in control of the car or could be a passenger. Although any movement can trigger cybersickness symptoms, particularly in those prone to motion sickness, there are specific factors that can influence this. For example, fast acceleration (al Zayer et al. 2019; Kim and Park 2020) and movement along the vertical axes (Kim and Park 2020; Pöhlmann et al. 2021) exacerbate cybersickness symptoms. In addition, movement in the periphery, as seen in global movement, is associated with more cybersickness than movement in the centre of the FOV (Lin et al. 2002; Ren et al. 2016). Finally, lags caused by motion‐to‐photon latency can increase cybersickness (Stauffert et al. 2020).

Global movement can be caused by the participant moving through the environment, and an important consideration is the way this movement occurs. Popular methods include walking‐based methods (e.g., natural walking or walking in place on an omnidirectional treadmill), teleportation (e.g., using controllers to indicate a target location and jumping or ‘teleporting’ to that location), controller‐based steering (e.g., a joystick or steering wheel) and spatial steering (e.g., gaze‐directed navigation) (Tian et al. 2022). There is evidence indicating that natural walking (Monteiro et al. 2018) and teleportation seem to cause less cybersickness than controller‐based or spatial steering (Saredakis et al. 2020; Tian et al. 2022). Walking naturally around the XR environment is limited by the physical space in the real world, including limitations placed by cables and environments required for neuroimaging (e.g., faraday cages and MSRs). Omnidirectional treadmills exist as an alternative mode; however, walking on these is much less natural with specific motion often needed to be carried out by the feet to propel the user through the environment. These can be problematic with some users reporting increased cybersickness (Chakraborty et al. 2024), which may be related to a lag between the movement the participant is making and the environment. When navigating the environment using controller‐based or spatial steering, it can be due to the participant using a virtual vehicle or can be an alternative to physical walking (treadmill or natural). In this situation, there may be more risk of cybersickness due to a mismatch between controller input and global movement in the XR environment. Teleportation, specifically without any animation of movement, has been shown to reduce the amount of sensory conflict and therefore cybersickness (e.g., Caputo et al. 2023; Moghadam et al. 2020; Tian et al. 2022, but also see Clifton and Palmisano 2019, for a more critical view). Taken together, mode of navigation can have a major impact on cybersickness.

In conclusion, researchers should carefully consider environment design to mitigate the risk of cybersickness and implement XR‐only practice sessions before introducing neuroimaging. For example, researchers may choose to limit movement of objects, use a reduced FOV or use teleportation for movement through a scene. This simplicity may seem like a limitation in experimental design; however, this reduces confounding factors related to cybersickness that may interfere with the research goals.

### 3.3 Realism and Complexity in Environment Design

One of the appeals of XR is its ability to present highly realistic scenarios, especially when compared to two‐dimensional devices such as screens or tablets. As researchers, we might be tempted to assume that we should strive for maximal realism in our XR environments, as this will maximise the ecological validity of our study. However, we argue against this for three reasons. First, substantial resources are required to design highly realistic XR environments, including expertise, time and computational resources. Second, realism can be detrimental, for example, increasing the realism of human and nonhuman virtual characters can result in a feeling of unease (Uncanny Valley effect; Wang et al. 2015). Third, the aim of XR studies is typically to evoke meaningful behaviours of interest, and we argue that realistic environments are not a prerequisite for this. This is demonstrated by a significant body of research which successfully elicited physiological and cognitive responses comparable to real world effects using limited graphics (Banakou et al. 2020; Draschkow et al. 2021; el Basbasse et al. 2023) and boundaries (room scale and stationary VR experiences, e.g., Alexander et al. 2024; el Basbasse et al. 2023; Gregory et al. 2022; Maoz et al. 2023).

Therefore, we propose researchers should focus on designing an environment that best elicits and captures the behaviour of interest. We argue that a key factor for this is how believable the environment is, that is, how cohesive and convincing the environment feels to the user. Together, there is a natural interaction between the goal of XR developers (increasing presence) and the goal of cognitive neuroscientists (observing ecologically valid behaviours).

Designing believable XR environments depends on a cohesive design, consistent physics and smooth, stable performance. Cohesive design refers to the use of stylistically similar elements. For example, it will disrupt the believability of an environment if a city consists of highly realistic buildings but uses cartoon cars. Consistent physics refers to any motion and behaviour of elements, including lighting. This is particularly important with regards to any moving objects (including users moving through the environment) and objects that users interact with. For example, any audio coming from elements in the virtual environment should be spatially and temporally consistent; a tree should cast a shadow that changes with the angle of the sun. Although not all physics properties of virtual environments need to be completely realistic, they need to be believable in the context of the environment and the experimental manipulations within it. Smooth and stable performance refers to the quality of the presentation. Any jitter or lag in the XR environment can disrupt behaviour and experience, including increased cybersickness. A highly realistic XR environment that constantly lags is less engaging and believable than a simple environment that runs smoothly.

To conclude, use of XR environments should add to the efficacy of the study, allowing us to understand the phenomenon under consideration better than a classic experimental study or a real‐world experience would.

## 4 Key Considerations for Using Neuro‐XR

In this article, we have outlined how XR technologies can be combined with neuroimaging technologies to gain insight into how the human brain processes realistic scenarios and tasks. This can increase the ecological validity of research allowing us to develop innovative research scenarios that can probe processes that were previously difficult to measure, such as body illusions (Matamala‐Gomez et al. 2021). In the previous sections, we presented three neuroimaging technologies (fNIRS, EEG and OP‐MEG) and discussed their XR compatibility. We also discussed design choices in XR environments and how these impact research outcomes. We will now critically consider the circumstances underpinning why you might use Neuro‐XR, ethical considerations and best practices.

### 4.1 Why Neuro‐XR?

The motivation for using XR methods in neuroscience assumes that the research question cannot be answered without them. From our position, we do not argue that this is a difficult assumption to meet. However, because both XR and neuroimaging can be challenging and costly to implement, it is important to consider these assumptions carefully. Here, we provide considerations that determine the suitability of XR and of neuroimaging methods for your research questions.

First, it is important to consider whether XR is a suitable medium to investigate your research question in a way that is different to screen‐based or real‐life methods. One aim of using XR for psychological research is to increase ecological validity by presenting three‐dimensional, dynamic and realistic stimuli. However, not all research questions depend on maximal ecological validity and using XR does not automatically guarantee ecological validity. For example, research on the fundamental cognitive mechanisms underlying task switching can be conducted successfully using simple lab‐based experiments (e.g., switching between categorising numbers as odd or even and categorising letters as vowels and consonants). Simply taking a typical lab‐based task switching paradigm, such as categorising letters or numbers, and conducting it in XR would not actually increase ecological validity, as the nature of the tasks themselves would not change. An advantage of Neuro‐XR lies in studying the brain under natural conditions, a primary motivation for cognitive neuroscientists. As such, it is most beneficial when the research question aligns with this focus.

Second, you should consider if applying XR‐compatible neuroimaging methods such as EEG, fNIRS or OP‐MEG is the best approach for your research question. The aim of using neuroimaging methods in research is to understand the underlying neural processes of a certain behaviour, and this requires a clear understanding of the measurement and interpretation of brain activity. Therefore, you should evaluate if you are truly interested in these neural processes or if other measures might be appropriate instead. For example, if broadly interested in task‐based attention, the behavioural data that can be collected in XR (including response times, body positions and movement) can already be informative on its own. In addition, cognitive processes such as attention or emotional arousal can also be measured using other psychophysiological methods such as recording skin conductance, eye movements or heart rate (Muhtadie et al. 2015). Importantly, these methods are less expensive as well as easier to implement and analyse than the neuroimaging methods we discussed here. If your research question does require neuroimaging methods, you then need to consider if the required study design can be realised using Neuro‐XR, while accounting for the issues raised above.

Third, you need to consider the population you are interested in understanding. Often studies involve neurotypical, young adults who are likely tolerant of XR and neuroimaging devices, yet there are still important considerations for this demographic. Regarding neuroimaging technology, it is important to be aware of systemic and structural racism issues whereby certain systems are not able to collect reliable data from participants with coarse, curly hair or darker skin tones. This has been discussed elsewhere (e.g., Choy et al. 2022; Parker and Ricard 2022; Webb et al. 2022), and although solutions are being explored (Etienne et al. 2020; Mlandu et al. 2024), it is important to be aware of how the technology used can be exclusionary. As a technique in its infancy, Neuro‐XR is well positioned to meet these challenges from the outset. Regarding XR, considerations should be made around the risk of cybersickness as well as the compatibility of XR devices with individual differences, for example, head size, religious headwear and vision differences.

Alternatively, if your research is with specialist populations, including children, older adults, neurodiverse individuals and clinical populations, there are additional factors to consider. For example, neuroimaging data obtained from children are typically noisier, a factor exacerbated by artefacts introduced by XR devices. Further, most HMDs are not suited for young children due to the devices' weight and size. In addition, some specialist populations might not tolerate certain Neuro‐XR equipment (e.g., due to sensory processing disorders). Therefore, we advise researchers to take the approach of patient and public involvement and engagement (PPIE) where the populations of interest are part of the study design. This allows the population of interest to both inform what the research is about and to inform if the research practice is inclusive of their needs.

Overall, Neuro‐XR is a novel technique that has ongoing developments across the different combinations. The phase of development varies and is dependent on the combination. First, EEG‐XR is the most well established and ready‐to‐use technique in its ability to perform as a reliable combination. This is reflected in the volume of papers published using EEG‐XR (Alexander et al. 2024; el Basbasse et al. 2023; Gregory et al. 2022; Huizeling et al. 2020; Senftleben and Kessler 2024) and, as a result, a community who can share best practices. As mentioned in Section 3.2, there are continued innovations to account for movement artefacts (Li and Huang 2025) and, therefore, room for improvement. Similarly, fNIRS‐XR is technically well suited to XR and has been paired successfully with HMD, AR and CAVE systems (Benitez‐Andonegui et al. 2020; Bulgarelli et al. 2023; Kim et al. 2019; Pinti et al. 2024). However, the evidence remains limited compared to EEG‐XR. The practical challenges are driven by movement artefacts (Yücel et al. 2021) and task design. Current analysis methods mostly rely on structured block or event‐related designs, which can be difficult to pair with naturalistic XR paradigms (Pinti et al. 2018). As a result, it is not yet possible to fully leverage the potential of the ecological validity of XR, leaving a mismatch between what fNIRS hardware can measure and what we can reliably analyse. Lastly, OP‐MEG‐XR does not provide an out‐of‐the‐box combination, and specific expertise, both in neuroimaging and XR, is needed to successfully create this pairing. Successful pairings have been documented (Roberts et al. 2019; Zabbah et al. 2026) and are becoming more streamlined but more work is needed to fully realise this combination as an accessible approach. Across all modalities, it becomes clear that the Neuro‐XR field still requires standardised procedures for data collection, noise management and data analysis, as well as a systematic investigation on how XR hardware affects neuroimaging signal quality. These limitations must be considered before deciding to use a Neuro‐XR approach.

## 5 Ethical Research Practices

An important consideration is the ethics of the research you are conducting. In research involving participants, the benefits of the research outcomes must outweigh the risks that participants might face. In the case of Neuro‐XR studies, there are physical, psychological and privacy aspects to consider.

Physical risks include risks caused by the XR equipment, such as cybersickness, as well as risks caused by the neuroimaging equipment, such as skin reactions to the gel used for EEG preparation. When taking appropriate measures to mitigate these risks (e.g., monitoring cybersickness symptoms), Neuro‐XR studies should not pose a big physical risk to participants in most cases.

Psychological risks include any adverse effects of a study on participants' mental status, such as emotional distress. This might be caused by exposing participants to upsetting stimuli (e.g., graphic scenes of accidents) or by asking participants to do something that makes them feel bad about themselves (e.g., making participants lie to other people). These risks can be present in any psychological study; however, the use of XR technology might increase their impact further (Ramirez 2019). This is because the sense of presence that is created by XR makes participants feel like they are truly experiencing the virtual environment. This could potentially lead to heightened emotional responses or distress upon re‐entry to reality (Slater et al. 2020). In addition, XR allows for more intense experimental manipulations, for example, manipulating participants' virtual avatars might cause feelings of body dysmorphia (Slater et al. 2020). Further, participants will often stay in uncomfortable situations because of a need to please the experimenter (i.e., demand characteristics and social desirability bias). Hence, a participant cannot be relied upon to say if they feel distressed. Therefore, we urge researchers to thoroughly assess their studies for any negative psychological effects they could cause through the use of national framework guidelines (e.g., UKRI's Framework for responsible research and innovation).

Privacy risks are centred around the rich data sets Neuro‐XR research can yield in the form of multidimensional and individualised profiles of participants. Although gaining such personalised data can be deemed a benefit to research, it also presents a heightened risk of removing anonymity. Recent work has shown that nonverbal data, such as body motion tracking, can be accurately identified (up to 95% success rate) by machine learning models (Miller et al. 2020). Nair et al. (2023) found similar evidence with over 50,000 participants' head and hand data that revealed a 94.33% accuracy from just 100 s of motion. This highlights the need for heightened data privacy considerations and explicit ethical recommendations for nonverbal data. With the current technological drive to produce wearables that are comprehensive in their data collection, analysis and output, we must consider the privacy risks that are simultaneously rising when choosing to use the technology.

Taken together, Neuro‐XR research can pose significant risks to participants. These risks need to be recognised in order to minimise and mitigate them. On a broader scale, the Neuro‐XR community should reflect on the ethical implications of their research with regards to societal questions and how they should be reviewed (e.g., see Kenwright 2018; Ramirez 2019; Slater et al. 2020; Spiegel 2018).

## 6 Best Reporting Practices

With any growing field, developing and maintaining a consistent and informative reporting of methodological aspects are critical to building a strong foundation. We therefore recommend that authors report the following in Neuro‐XR papers and when sharing data sets: headset refresh rate; controller or tracking sampling rate; synchronisation method; validation of event triggers; movement constraints; cybersickness monitoring; and artefact handling procedures. We also recommend that although data sharing is good practice, and often expected by funders and journals, authors should carefully consider what is appropriate to share given privacy concerns outlined above. For example, data should be stripped of identifying metadata, and only the elements of the data required for analysis should be considered for sharing, with this also reviewed for privacy risk.

## 7 Future Outlook

XR technology is rapidly advancing and becoming widely available. In turn, innovations in the field of XR will provide more opportunities for Neuro‐XR research, potentially revolutionising the fields of psychology and cognitive neuroscience. This also transfers to applied fields such as brain‐computer interfaces and clinical settings. Making use of these innovations depends on open‐source tools for XR research, be that experimental frameworks, environment design or measurement solutions (see Table S1). It should be a key priority to maintain, grow and sustain such solutions. This can only be achieved through neuroimaging and XR users coming together to share their knowledge and best practices. Such an emerging Neuro‐XR community should aim to also include voices from other fields such as educators, artists and clinicians to truly push the field of Neuro‐XR forward. These collaborations will foster the design and application of advancements in the field (e.g., multiuser, room‐scale XR, signal processing pipelines and designing XR equipment specifically for neuroscience). In addition, the Neuro‐XR community needs to determine and uphold standards for ethical and research practice to ensure the reliability and robustness of findings (Lanier et al. 2019). By doing so, Neuro‐XR has the promise to deliver impactful innovative research that bridges the gap between the lab and the real world.

## 8 Conclusion

Overall, we believe these combinations can be a powerful tool in research settings, as they provide the opportunity to ask questions that cannot be answered with traditional set‐ups; however, it is important that researchers are thoughtful in their motivation and design of Neuro‐XR experiments. Collectively, we must share and update ethical considerations that are unique to the developing technologies while maintaining a push for open science. Collaboration is central to the success of Neuro‐XR and should be prioritised as a community.

## Author Contributions

**Clíona L. Murray:** conceptualization, writing – review and editing, writing – original draft, visualization, project administration. **Nicholas A. Alexander:** writing – review and editing, writing – original draft. **Paola Pinti:** writing – original draft, writing – review and editing. **Samantha E. A. Gregory:** conceptualization, writing – original draft, writing – review and editing. **Ulrike Senftleben:** conceptualization, writing – original draft, writing – review and editing.

## Funding

This work was supported by the Yale Science Fellowship, the Yale Kavli Institute for Neuroscience and the Wellcome Trust (226793/Z/22/Z and 212979/Z/18/Z).

## Conflicts of Interest

The authors declare no conflicts of interest.

## Supporting information


**Table S1:** Translated and adapted from HAN Ming, KUAI Shu‐Guang (2024). Toolkits for virtual reality research in psychology. Advances in Psychological Science, 32(11), 1800–1813. See also https://ieeexplore.ieee.org/stamp/stamp.jsp?tp=&arnumber=9599343.

## Data Availability

Data sharing is not applicable to this article, as no datasets were generated or analysed during the current study.
