# Supplementary material for: Neuro‐XR Primer: Blending Neuroscience and Extended Reality to Facilitate Cognitive Investigation
Source: Eur J Neurosci. 2026 Oct 28;64(8):e70733. doi: 10.1111/ejn.70733 (PMC13716529; doi:10.1111/ejn.70733)
Supplement: Supplementary file 1 [file EJN-64-0-s001.pdf]

Supplementary Material

Table S1

Translated and adapted from HAN Ming, KUAI Shu-Guang. (2024). Toolkits for virtual reality research in psychology. Advances in Psychological Science, 32(11), 1800-1813. See also https://ieeexplore.ieee.org/stamp/stamp.jsp?tp=&arnumber=9599343

Note that most solutions require researchers to be using Unreal (https://www.unrealengine.com/en-US), Unity (https://unity.com/) or Vizard (https://www.worldviz.com/vizard-virtual-reality-software) to host their studies. Unreal and Unity are gaming engines that are free to use non-commercially. Vizard is a python based VR development platform with free and paid versions.https://github.com/seckincengiz/xr

Tool	Description	Source Reference	Paper/DOI	Dependencies (Programming language)
**Platforms**				
Unity Engine	Programme to create interactive virtual reality environments.	https://unity.com/products/unity-engine		None (C#)
Unreal Engine	Programme to create interactive virtual reality environments.	https://www.unrealengine.com/en-US		None (C++ and Blueprints)
Vizard	Programme to create interactive VR applications for research	https://www.worldviz.com/vizard-virtual-reality-software		None (Python)
**Experimental Frameworks**				
UXF	General framework to create core modules for experiments through structured frameworks	http://github.com/immersivecognition/unity-experiment-framework	https://doi.org/10.3758/s13428-019-01242-0	Unity Engine (C#/ Java Script)

bmlTUX	General framework for designing experiments with no code	https://biomotionlab.github.io/TUX/	https://doi.org/10.1177/2041669520938400	Unity Engine (C#/ Java Script)
EVE Framework	A content-specific framework with core features to develop navigatable environments.	https://cog-ethz.github.io/EVE/	https://doi.org/10.1007/978-3-319-68189-4_10	Unity Engine (C#/ Java Script/ SQL)
Landmarks	A content-specific framework for developing experiments on spatial navigation	https://github.com/mjstarrett/Landmarks	https://link.springer.com/article/10.3758/s13428-020-01481-6	Unity Engine (C#)
OpenMaze	A content specific framework for spatial navigation experiments, configurable through files	https://github.com/DuncanLab/OpenMaze/releases/tag/v1.1.0	https://doi.org/10.3758/s13428-021-01664-9	Unity Engine (C#)
PTVR	A content-specific framework that uses a visual perception experiment tool	https://ptvr.inria.fr/	https://doi.org/10.1167/jov.24.4.19	Unity Engine (Supports Python 3.6)
Study Framework Plugin	General framework for the set up and execution of factorial-design studies in Unreal Engine	https://git-ce.rwth-aachen.de/vr-vis/VR-Group/unreal-development/plugins/unreal-study-framework	https://doi.org/10.1109/VRW62533.2024.00087	Unreal Engine 4.X (C++)
**Environments and assets**				
VRthreat Toolkit	Contains naturalistic environments initially designed for creating threat scenario experiments	https://xip.uclb.com/product/vrthreat-toolkit-for-unity	https://doi.org/10.3758/s13428-023-02241-y	Unity Engine (C#/ Java Script)

Blender	A 3D computer graphics software tool set that can be used to create animated films, visual effects, art, 3D-printed models, motion graphics, interactive 3D applications and virtual reality.	https://www.blender.org/		None
DomeVR	Tools for creating virtual environments for experiments involving humans or animals	https://github.com/zero-noise-lab/DomeVR	https://pmc.ncbi.nlm.nih.gov/articles/PMC11737658/	Unreal Engine 4.X (C++)
Hafnia Hands	Various hand models for VR studies, including diverse skin tones and non-human models	https://github.com/henningpohl/hafnia-hands	https://www.frontiersin.org/article/10.3389/frvir.2022.719506	Unity Engine
Make Human	A 3D computer graphics middleware designed for the prototyping of photorealistic humanoids.	https://static.makehumancommunity.org/		None
Mixamo	3D characters, skeletal rigs and animations, ready for use in films, games, interactive experiences and illustration	https://www.mixamo.com/#/		None
NavWell	Web-based design for virtual water maze tasks, navigation experiments in Unity	https://navwell.cs.nuim.ie/home	https://doi.org/10.3758/s13428-019-01310-5	Unity Engine (C#/HTML)
OSAHL	3D Black avatars created by Black artists/ FBX format	https://afrohairlibrary.org/		None
The CogLearn Toolkit	A virtual reality platform to investigate avoidance learning in naturaliastic settings, using an uncomfortable sound, a physically plausible avoidance		https://doi.org/10.3758/s13428-025-02630-5	Unity Engine (C#)

	action and allowing for unconstrained movements.			
VALID	FBX format 3D avatars with diverse skin colors and validated ethnic backgrounds, including skeletal rigging	https://github.com/xrtlab/Validated-Avatar-Library-for-Inclusion-and-Diversity---VALID	https://www.frontiersin.org/articles/10.3389/frvir.2023.1248915	None
**Measurement toolkits**				
VNT	Contains tools to implement and run spatial navigation experiments	https://gitlab.ub.uni-bielefeld.de/virtual_navigation_tools/unity_vnt_main	https://doi.org/10.1371/journal.pone.0293536	Unity Engine (C#)
Excite-O-Meter	Records and analyzes heart rate variability data in Unity	https://sites.google.com/view/exciteometer/		Unity Engine (JavaScript)
ExpyVR	Modified PsychoPy script for designing and running experimenys in VR	https://www.epfl.ch/labs/lnco/research/expyvr/		None (Python/ C/ C++)
IXCI	Web-based interface for controlling and monitoring Unity VR programs	https://github.com/alreylz/ixci	https://dl.acm.org/doi/10.1145/3474349.3480217	Unity Engine (C#/ JavaScript/ HTML)
MR-RIEW	Toolkit for designing experiment workflow including instructions, environments, sessions, trials and questionnaires	https://github.com/CrowdVRLab/MR-RIEW?tab=readme-ov-file	https://ieeexplore.ieee.org/document/9757432	Unity Engine (C#/ Java Script)
PARE-VR	Heart rate variability measurement using a wearable device (smartwatch, smartphone) integrated with Unity	https://github.com/luisqtr/pare-vr	https://doi.org/10.1109/AIVR46125.2019.00027 https://doi.org/10.1109/ICDMW.2019.00123	Unity Engine (C#/ C++/ Java)

QuickVR	A library based on Unity which not only offers embodiment in a virtual character, but also provides a series of high level features that are necessary in any VR application	https://gitlab.com/eventlabprojects/quickvr.packages/com.quickvr.quickbase	https://www.frontiersin.org/journals/virtual-reality/articles/10.3389/frvir.2022.937191/full	Unity Engine (C#/ Java Script)
R2VR	Implements and runs VR experiments in R	https://github.com/ACEMS/r2vr		None (R)
vexptoolbox	Facilitates the development of experiments on the Vizard platform	https://github.com/ischtz/vizard-experiment-toolbox	https://link.springer.com/article/10.3758/s13428-022-01831-6	Python
VRQuestionnaireToolkit	Tool for conducting surveys in virtual reality environments	https://github.com/MartinFk/VRQuestionnaireToolkit	https://dl.acm.org/doi/10.1145/3379350.3416188	Unity Engine (C#/ Java Script)
VRSTK	Multifunctional development framework, supports integration with other plugins for various functionalities	https://github.com/ixperience-lab/VRSTK		Unity Engine (C# /R /Python /Java Script)
